# Analysis of factors influencing HPV vaccination intention among Chinese college students: structural equation modeling based on health belief theory

**DOI:** 10.3389/fpubh.2024.1510193

**Published:** 2025-01-30

**Authors:** Shi-Yuan Song, Ying Guo, Yi-Hua Li, Zheng Wang, Wei Gao

**Affiliations:** ^1^Department of Epidemiology, Preventive Medicine, Medical School, Yanbian University, Yanji, China; ^2^School Hygiene and Disinfection Products Hygiene Supervision Section, Yanji City Center for Disease Control and Prevention, Yanji, China

**Keywords:** HPV vaccine, vaccination intentions, health belief model, structural equation modeling, minority area

## Abstract

**Background:**

Increasing human papillomavirus (HPV) vaccination rates is vital for achieving society-wide public health goals, yet current research on HPV vaccine-related knowledge, vaccination intentions, and behaviors among college students in ethnic minority regions is inadequate. This investigation sought to explore the present circumstances of college students in China’s ethnic minority regions concerning their awareness, attitudes, and practices related to the HPV vaccine. This study also aimed to provide a scientific basis for future health education and HPV vaccine promotion in China’s college student population.

**Methods:**

Based on health belief theory, 1,388 valid questionnaires were collected online to investigate college students’ knowledge, beliefs, and behaviors related to HPV vaccination and the factors influencing their willingness to be vaccinated. The data were analyzed via SPSS 26.0 for descriptive analysis and Amos 24.0 for structural equation modeling, factor analysis and path analysis.

**Results:**

The overall HPV vaccine awareness rate was 77.9%. In terms of health attitudes, the positive intention rate was 50.4%, with females having more positive attitudes than males (OR = 2.242, 95% CI = 1.777–2.829). In terms of health behaviors, the rate of positive behaviors was low (40.0%), and the probability of positive behaviors was significantly lower for students with nonmedical-related majors than for those with medical majors (OR = 0.579, 95% CI = 0.442–0.759). The results of the structural equation modeling analysis revealed that college students’ perceptions of the perceived benefits of the HPV vaccine positively and directly affect their willingness to be vaccinated (*β* = 0.290, *p* < 0.001), and perceived severity has an indirect effect on their willingness to be vaccinated (*β* = 0.198, *p* < 0.05).

**Conclusion:**

Although college students in ethnic minority areas have a high rate of HPV-related knowledge, their willingness to be actively vaccinated and their positive behaviors need to be improved. In addition, enhancing the levels of perceived severity and perceived efficacy among college students can help increase their willingness to receive the HPV vaccine.

## 1 Introduction

Cervical cancer is the 4th most common malignant tumor threatening the health of the female reproductive system after breast, colorectal, and lung cancers (1). It is estimated that more than 58% of global cervical cancer cases occur in Asia, of which 18% occur in China (2). In 2020, there were 110,000 new cases of cervical cancer in China, with 60,000 fatalities (3). This makes the disease a major public health concern that endangers health of women in the country. Reports indicate a close relationship between high-risk human papillomavirus infection and the occurrence of cervical cancer (4). Sexual contact, including vaginal intercourse, oral sex, and anal sex, primarily transmits HPV and is a necessary condition for cervical cancer. Therefore, preventing HPV infection has become a key measure to reduce the risk of cervical cancer. In recent years, a number of studies have confirmed that vaccination against HPV vaccination can effectively prevent such infection, thus significantly reducing the risk of cervical cancer (5, 6). Studies have suggested that vaccination rates can reach more than 75% in some high-level-income countries; however, many countries still have low vaccination rates (6).

In China, the knowledge and vaccination rate of the HPV vaccine among school-aged college students in inland areas are generally low, with only 46.7% of college students having a good level of knowledge of cervical cancer prevention and treatment (7) and only 10.4% of students indicating that they have received the HPV vaccine (8). As a group with a high level of education, college students have more autonomy in terms of whether to receive the HPV vaccine (9). Studies have also confirmed that 68.8% of students regard a university education as one of the main ways to obtain information about vaccines (10). Therefore, the level of knowledge, willingness, and behavior of vaccination during university years plays an important role in promoting the popularization of HPV vaccines. In addition, premarital sex among college students is increasing, further exacerbating the potential risk of HPV infection. Understanding the level of HPV knowledge among college students and its impact on their vaccination willingness and behavior is of great public health significance. Factors are of enormous significance for the prevention and control of cervical cancer in China.

A variety of demographic factors may influence HPV knowledge, vaccination willingness, and vaccination behavior. Research indicates that various factors such as gender (11), grade (11), ethnicity (12), major (13), financial burden of vaccine costs (14), concerns about vaccine side effects (14), sexual behavior (14), and whether family members have a history of cervical cancer (12) significantly influence HPV-related knowledge, vaccination willingness, and vaccination behavior. Therefore, exploring the differences in HPV-related knowledge, willingness, and behavior among college students based on demographic factors is of great significance for formulating targeted strategies to promote cervical cancer prevention and control.

The health belief model (HBM) is a prevalent theoretical framework for evaluating and forecasting individual health behaviors. The model posits that an individual’s readiness to engage in preventive health behaviors through both direct and indirect pathways (15). Perceived susceptibility, perceived severity, perceived barriers, and perceived benefits directly influence the direct pathway, which in turn influences the implementation of health behaviors. Perceived susceptibility refers to an individual’s subjective assessment of the risk of a health problem or disease occurring; perceived severity refers to an individual’s subjective judgment of the possible harmful consequences of the health problem or disease; perceived barriers refers to the obstacles or difficulties an individual faces in adopting a health behavior; perceived benefits refers to the positive effects or benefits an individual believes may result from adopting a particular health behavior; and perceived benefits refers to the positive effects or benefits an individual believes may result from adopting a particular health behavior. Perceived barriers refers to the obstacles or difficulties faced by individuals in adopting health behaviors; perceived benefits refers to the positive impacts or benefits that individuals believe may be brought about by adopting specific health behaviors. Perceived severity indirectly influences the willingness to engage in health behaviors through mediating variables like perceived benefits or self-efficacy, forming the indirect pathway. Therefore, the health belief model not only focuses on direct cause-and-effect relationships but also reveals the complex interactions between constructs. In recent years, the HBM has shown to be a valid and reliable tool for assessing health beliefs, and it has been used in studies related to HPV vaccination intentions in several regions. A previous study in Italy showed that students are more likely to complete the vaccination program when they have a greater awareness of the benefits of the HPV vaccine (16); other studies have also indicated that there is a direct relationship between willingness to vaccinate, perceived sensitivity, and self-efficacy, with a negative correlation with perceived handicap (17). Thus, good health behaviors under the HBM framework are crucial for increasing the willingness to receive HPV vaccination and vaccine coverage and thus improving the level of public health awareness (18–20).

Studies have shown that due to cultural background, lifestyle, and socioeconomic conditions, the health awareness and behaviors of people in minority areas often differ significantly from those in other areas (21–23). In addition, medical resources in minority areas are relatively limited (24), and the health literacy rate is low, which may further affect the awareness and willingness to vaccinate against HPV.

The institution examined is situated in Yanbian Korean Autonomous Prefecture, a region in China characterized by a minority population, and operates as a full-time comprehensive public university. It is one of the colleges and universities previously established by the Communist Party of China in ethnic minority areas; thus, it has a large population of ethnic minority students and strong ethnic regional characteristics. Furthermore, it possesses the typical attributes of a comprehensive university and exhibits significant universality and representativeness, and can better represent the characteristics of the group of college students from minority universities in China. Thus, investigating the positive level of college students’ knowledge, attitudes, and behaviors related to the HPV vaccine at this university is conducive to further understanding the status of college students in ethnic minority colleges and universities in China regarding HPV vaccination and its influencing factors in carrying out health education in a targeted manner.

Although in recent years, there have been a large number of studies on the factors affecting the willingness of Chinese college students to receive the HPV vaccine. However, most of the existing studies focus on urban areas or mainstream groups (10, 25–27), and there is limited research on the willingness of college students in ethnic minority areas to receive the HPV vaccine. On the basis of the above background, the current study aimed to not only investigate the knowledge, willingness to vaccinate, and positive behaviors of college students in ethnic minority areas in China with respect to the HPV vaccine and the related influencing factors, in order to fill the research gap in this field. In addition, previous studies have mainly focused on the analysis of a single factor, while this study is based on the Health Belief Model, which comprehensively considers the impact of multiple factors on vaccination intentions. It uses a structural equation model to conduct an in-depth analysis, so as to provide a scientific basis for formulating more targeted vaccine promotion strategies and for future health education and HPV vaccine promotion among college students from minority groups in China.

## 2 Materials and methods

### 2.1 Study population and data collection

The target population of this study is full-time college students at a university in an ethnic minority area in China. Considering the limitations of the actual situation at the implementation stage of the sample survey, nonprobability sampling (convenience sampling) was used for the present investigation (27, 28). Nonprobability sampling transpires when the researcher chooses a subject for which information is readily accessible, reflecting the current circumstances. This implies that increasing the sample size can mitigate data bias stemming from individual differences, thereby enhancing the credibility and validity of the research findings. As a result, the survey collected data from a large sample of 1,388 respondents, with a valid response rate of 100%. There were no missing values in this study.

The online platform “Questionnaire Star” was used to create an electronic questionnaire, a presurvey was conducted (300 people), and after the questionnaire was refined, the investigators conducted a formal face-to-face survey from May 27 to June 27, 2024. The criteria for including study subjects were as follows: (1) full-time college students aged 18–26 years and (2) obtaining informed consent for participation in this research study and voluntary involvement in this survey. The exclusion criteria were delineated as follows: (1) people with allergic reactions to vaccines and (2) patients who were diagnosed with severe cognitive disorders.

This study set individuals aged 18–26 as the target population for the study, based on the following considerations: individuals in this age group are usually in an important period of sexual health education, and HPV vaccination still has a high preventive benefit. In addition, excluding individuals who have been diagnosed with severe cognitive impairment is to ensure that the questionnaire can be accurately understood and completed, thereby reducing potential data bias and improving the scientific nature of the research results.

### 2.2 Survey instruments and quality control

#### 2.2.1 General information questionnaire

These factors include gender, ethnicity, urban/rural status, specialty, grade level, monthly living expenses, parents’ education level, history of cervical cancer in family members or close friends, and history of sexual behavior.

#### 2.2.2 Self-administered HPV and HPV vaccination knowledge and beliefs questionnaire

The questionnaires used to assess HPV-related knowledge, willingness, and behaviors were designed based on previous studies and authoritative guidelines (29–31) and were finalized under expert review and guidance. This questionnaire consisted of 28 questions. There were 15 questions on HPV-related knowledge, all of which were single-choice, with one point given for a correct answer and no points given for an incorrect answer, for a maximum possible total of 15 points. The HPV vaccination willingness questionnaire had nine questions. A 5-point Likert scale was used to assign scores to the nine entries (very reluctant/strongly disagree = 1 point, very willing/strongly agree = 5 points), with a maximum possible score of 45 points, The Cronbach’s *α* for this part of the scale was 0.924, indicating that it has good internal consistency. HPV vaccine-related behaviors were assigned a score (yes = 1 point, no = 0 points) for four questions, with a maximum possible score of 4 points. To more clearly distinguish between different levels of cognition, willingness, and behavior, this study divided continuous variables into dichotomous variables during data analysis (12, 14). We used the 80% quartile as a threshold and the percentile as a criterion for division. Health-related research has widely used this method (12, 28). In this study, a score of ≥12 on the knowledge level was defined as a high-awareness situation, with 80% used as the cutoff, and this population was defined as the aware population; a score of ≥36 on the health intention dimension was defined as positive intention; and a score of ≥3 on the health behavior dimension was defined as positive behavior. This dichotomous categorization helps classify the study population into high- and low-risk categories, which facilitates more targeted intervention designs for high- and low-risk groups at a later date.

#### 2.2.3 Health beliefs scale

The Health Belief Model (HBM)-based study used structural equation modeling (SEM) to analyze the direct and indirect pathways of influence of the constructs (perceived benefits, perceived severity, perceived barriers, self-efficacy, etc.) on vaccination intentions. The main goal of SEM modeling is to explore the relationships among the core constructs of the HBM theory as well as their pathways of influence. Therefore, we limited the scope of the SEM analysis to the Health Belief Model’s theoretical framework and excluded demographic variables. In descriptive and logistic regression analyses, demographic variables had big impacts on HPV knowledge, vaccination intentions, and behaviors. However, these impacts did not directly show how the HBM constructs were related to each other. Therefore, to avoid overly complex models or deviation from the HBM theoretical framework, we did not include demographic variables in the SEM model.

Therefore, this section extracts model entries of health beliefs associated with HPV vaccination through a systematic review of previous studies (32–35). Five dimensions were examined in terms of perceived susceptibility (2 entries), perceived severity (5 entries), perceived handicap (3 entries), self-efficacy (3 entries), and perceived benefit (3 entries). Each question was scored on a 5-point Likert scale ranging from “strongly disagree” to “strongly agree,” with higher scores indicating stronger beliefs about the individual’s health (the “perceived obstructiveness” section was reverse scored, with “strongly disagree” scored as 5 and “strongly agree” scored as 1). We invited two epidemiologists to evaluate the applicability and scientific validity of the entries’ content, and we conducted a pre-test among 300 students. The Cronbach’s alpha values for the five dimensions of the scale were 0.716, 0.912, 0.779, 0.791, and 0.959, all of which indicated good reliability.

### 2.3 Data analysis methods

SPSS 26.0 software and Amos 24.0 software were used to organize and analyze the data. Descriptive analyses of the respondents were carried out via SPSS, and count data were expressed as component ratios. The influencing factors were examined using one-way analysis of variance and binary logistic regression. Structural equation modeling (SEM) via Amos was used for confirmatory factor analysis (CFA), path analysis, and mediation tests. A two-sided test was used, with a test level of *α* = 0.05.

### 2.4 Ethical reflections

Informed consent was secured from all study participants; they were apprised of their right to withdraw from the study and assured that their data would remain strictly confidential and utilized solely for scientific analysis. The research was executed in accordance with the principles of the Declaration of Helsinki and received approval from the Institutional Review Board of Yanbian University (Ethics Code: 10187).

## 3 Results

### 3.1 General demographic characteristics

This study distributed 1,388 questionnaires, receiving 1,388 valid responses, resulting in a validity rate of 100%. Male and female students accounted for 38.83% (539) and 61.17% (849) of the sample, respectively. The vast majority of the students were Han Chinese (76.37%), with the type of residence being urban (72.05%) and nonmedically related (76.08%). A total of 30.12% were in their freshman year, and 26.01% were in their sophomore year; the monthly living expenses were predominantly 1,000–1999 RMB (47.41%). The literacy level of both parents was predominantly middle school (30.33, 29.32%). The vast majority of the students had family members who did not practice a medical-related profession (79.32%), had no family members with cervical cancer (89.19%), and were not sexually active (83.36%). The distribution of basic details is shown in Table 1.

**Table 1:** General demographic characteristics of survey respondents.

Participants		Frequency (n)	Percent (%)
Gender	Male	539	38.83
	Female	849	61.17
Ethnic group	Han	1,060	76.37
	Korean	202	14.55
	Other nationality	126	9.08
Town and country	Town	1,000	72.05
	Country	388	27.95
Major	Medical major	332	23.92
	Non-medical major	1,056	76.08
Grade	Freshman	418	30.12
	Sophomore	361	26.01
	Junior	287	20.68
	Senior	111	8.00
	Five-grade	14	1.01
	First-year graduate students	100	7.20
	Second-year graduate student	52	3.75
	Third-year graduate student	40	2.88
	Doctoral student	5	0.36
Monthly living expenses	<1,000	58	4.18
	1,000–1,999	658	47.41
	2,000–2,999	539	38.83
	3,000–3,999	87	6.27
	≥4,000	46	3.31
Father’s education level	Illiteracy	25	1.80
	Secondary school	163	11.74
	Junior high school	421	30.33
	Senior high school	285	20.53
	Specialized training school	190	13.69
	Undergraduate	244	17.58
	Graduate student and above	60	4.32
Mother’s education level	Illiteracy	35	2.52
	Secondary school	209	15.06
	Junior high school	407	29.32
	Senior high school	284	20.46
	Specialized training school	174	12.54
	Undergraduate	221	15.92
	Graduate student and above	58	4.18
Family members in medical-related professions	Yes	287	20.68
	No	1,101	79.32
Family member with cervical cancer	Yes	30	2.16
	No	1,238	89.19
	Currently unknown	120	8.65
Sexually active	Yes	231	16.64
	No	1,157	83.36

### 3.2 Current status of HPV in college students

#### 3.2.1 Knowledge of HPV-related knowledge among college students

The total knowledge rate of the 1,388 university students with regard to HPV was 77.9%, and the mean knowledge score was 12.44 ± 2.237, which suggests an excellent overall knowledge rate. The knowledge rates of the different questions ranged from 39.99 to 93.66%, as shown in Table 2. Knowledge of the entry “HPV types 6 and 11 are not high-risk types” was the lowest, at 39.99%. While the knowledge rates of the “HPV infection may be asymptomatic” (60.73%) and “Man can also be vaccinated against HPV” (75.36%) entries were low, the knowledge rates of the remaining entries were good.

**Table 2:** Knowledge about HPV among college students in higher education.

Item	Number of people aware (n)	Awareness rate (%)
HPV infection may have no symptoms	843	60.73
HPV infection can cause diseases other than cervical cancer	1,237	89.12
HPV can be detected through screening	1,222	88.04
Proper condom use reduces HPV infections	1,291	93.01
Men do not get HPV	1,272	91.64
If you have never had sex, you do not have a chance of getting cervical cancer	1,265	91.14
HPV types 16 and 18 are high-risk types	1,193	85.95
HPV types 6 and 11 are not high-risk types	555	39.99
Do you know the meaning of “price”?	1,250	90.06
HPV vaccination can help prevent cervical cancer	1,288	92.80
Cervical screening is still needed after HPV vaccination	1,300	93.66
The best time to get the HPV vaccine is before first sex	1,184	85.30
HPV Vaccination Prevents 100% Cervical Cancer	1,119	80.62
The nine-valent vaccine protects against more types of HPV than the bivalent and quadrivalent vaccines	1,196	86.17
Men can also get the HPV vaccine	1,046	75.36

The overall rate of knowledge about HPV was good, with 77.95% of the total number of people being considered qualified in terms of knowledge. The results of the univariate analysis revealed differences in HPV-related knowledge among college students of different genders, ethnicities, majors, grades, monthly living expenses, parental literacy, family history of cervical cancer, and sex status. The results are shown in Table 3. Among them, the rate of awareness was found to be significantly higher among women than among men; the knowledge rate of Han Chinese was determined to be markedly superior to that of other ethnic groups; the awareness rate was found to be higher among students with medical-related majors than among nonmedical students; knowledge rates were found to be higher among first-year research students than among students in the remaining grades; students with a monthly living wage of 2,000–2,999 were found to be more aware than the remaining class-living wage students; the knowledge rate of students whose parents’ education level is specialized was found to be higher than that of students whose parents have other academic qualifications; and knowledge rates were found to be highest among students whose family members did not have cervical cancer (all *p* values <0.05).

**Table 3:** Univariate analysis of knowledge about HPV among college students with different demographic characteristics.

Participants		Number of people aware (%)	χ^2^	p	Partial eta squared
Gender	Male	383 (71.06)	24.386	<0.001*	0.018
	Female	699 (82.33)	–	–	–
Ethnic group	Han	854 (80.57)	18.684	<0.001*	0.013
	Korean	137 (67.82)	–	–	–
	Other nationality	91 (72.22)	–	–	–
Town and country	Town	781 (56.27)	0.044	0.833	0.000
	Country	301 (21.69)	–	–	–
Major	Medical major	278 (83.73)	8.486	0.004*	0.006
	Non–medical major	804 (76.14)	–	–	–
Grade	Freshman	323 (77.27)	16.020	0.042*	0.012
	Sophomore	270 (74.79)	–	–	–
	Junior	218 (75.96)	–	–	–
	Senior	93 (83.78)	–	–	–
	Five–grade	12 (85.71)	–	–	–
	First–year graduate students	86 (86.00)	–	–	–
	Second–year graduate student	44 (84.62)	–	–	–
	Third–year graduate student	34 (85.00)	–	–	–
	Doctoral student	2 (40.00)	–	–	–
Monthly living expenses	<1,000	34 (58.62)	22.571	<0.001*	0.016
	1,000–1,999	519 (78.88)	–	–	–
	2,000–2,999	436 (80.89)	–	–	–
	3,000–3,999	64 (73.56)	–	–	–
	≥4,000	29 (63.04)	–	–	–
Father’s education level	Illiteracy	13 (52.00)	23.108	<0.001*	0.017
	Secondary school	130 (79.75)	–	–	–
	Junior high school	336 (79.81)	–	–	–
	Senior high school	215 (75.44)	–	–	–
	Specialized training school	159 (83.68)	–	–	–
	Undergraduate	191 (78.28)	–	–	–
	Graduate student and above	38 (63.33)	–	–	–
Mother’s education level	Illiteracy	19 (54.29)	35.135	<0.001*	0.025
	Secondary school	165 (78.95)	–	–	–
	Junior high school	321 (78.87)	–	–	–
	Senior high school	224 (78.87)	–	–	–
	Specialized training school	143 (82.18)	–	–	–
	Undergraduate	179 (81.00)	–	–	–
	Graduate student and above	31 (53.45)	–	–	–
Family members in medical–related professions	Yes	224 (16.14)	0.002	0.965	0.000
	No	858 (61.82)	–	–	–
Family member with cervical cancer	Yes	16 (53.33)	14.389	<0.001*	0.010
	No	980 (79.16)	–	–	–
	Currently unknown	86 (71.66)	–	–	–
Sexually active	Yes	176 (12.68)	0.501	0.479	0.000
	No	906 (65.27)	–	–	–

*p < 0.05.

When we looked at the variables that were significantly different in the univariate analysis, we observed that being female and having a high level of knowledge about HPV were linked (OR = 1.824, 95% CI = 1.389–2.395). Moreover, there was an inverse relationship found between Korean ethnicity and a good level of knowledge compared with the findings for Han Chinese ethnicity (OR = 0.527, 95% CI = 0.368–0.754). There was an inverse relationship found between the population of nonmedically related specialties and knowledge (OR = 0.589, 95% CI = 0.415–0.835). In addition, there was a positive association found between those with monthly living expenses of 2,000–2,999 RMB and a good level of HPV knowledge compared with those with monthly living expenses of <1,000 RMB (OR = 1.901, 95% CI = 1.008–3.585). There was a positive relationship found between those whose family members did not have cervical cancer and knowledge (OR = 3.094, 95% CI = 1.414–6.770), as shown in Table 4.

**Table 4:** Multifactorial logistic regression analysis of HPV-related knowledge among college students in higher education.

Participants		*β* value	SE value	Wald χ^2^ value	*p* value	OR value (95%CI)
Gender	Male	–	–	–	–	1.00
	Female	0.601	0.139	18.677	<0.001*	1.824 (1.389 ~ 2.395)
Ethnic group	Han	–	–	–	–	1.00
	Korean	−0.641	0.183	12.281	<0.001*	0.527 (0.368 ~ 0.754)
	Other nationality	−0.418	0.228	3.344	0.067	0.659 (0.421 ~ 1.03)
Major	Medical major	–	–	–	–	1.00
	Non–medical major	−0.529	0.178	8.832	0.003*	0.589 (0.415 ~ 0.835)
Monthly living expenses	<1,000	–	–	–	–	1.00
	1,000–1,999	0.559	0.314	3.172	0.075	1.748 (0.945 ~ 3.233)
	2,000–2,999	0.642	0.324	3.935	0.047*	1.901 (1.008 ~ 3.585)
	3,000–3,999	0.531	0.4	1.757	0.185	1.700 (0.776 ~ 3.725)
	≥4,000	0.228	0.46	0.245	0.621	1.256 (0.510 ~ 3.091)
Father’s education level	Illiteracy	–	–	–	–	1.00
	Secondary school	0.3	0.574	0.273	0.601	1.350 (0.438 ~ 4.161)
	Junior high school	0.158	0.563	0.079	0.779	1.172 (0.389 ~ 3.533)
	Senior high school	0.299	0.577	0.268	0.605	1.348 (0.435 ~ 4.176)
	Specialized training school	−0.035	0.595	0.003	0.954	0.966 (0.301 ~ 3.103)
	Undergraduate	−0.021	0.596	0.001	0.972	0.980 (0.305 ~ 3.148)
	Graduate student and above	0.755	0.687	1.208	0.272	2.128 (0.553 ~ 8.188)
Mother’s education level	Illiteracy	–	–	–	–	1.00
	Secondary school	0.016	0.493	0.001	0.973	1.017 (0.387 ~ 2.673)
	Junior high school	−0.558	0.483	1.338	0.247	0.572 (0.222 ~ 1.474)
	Senior high school	−0.535	0.499	1.152	0.283	0.586 (0.220 ~ 1.556)
	Specialized training school	−0.814	0.519	2.46	0.117	0.443 (0.160 ~ 1.225)
	Undergraduate	−0.821	0.52	2.493	0.114	0.440 (0.159 ~ 1.219)
	Graduate student and above	−1.117	0.629	3.152	0.076	0.327 (0.095 ~ 1.123)
Family member with cervical cancer	Yes	–	–	–	–	1.00
	No	1.13	0.399	7.995	0.005*	3.094 (1.414 ~ 6.770)
	Currently unknown	0.817	0.447	3.332	0.068	2.263 (0.942 ~ 5.439)

*p < 0.05.

#### 3.2.2 College students’ willingness to be vaccinated against HPV

The total positive willingness rate of college students to receive the HPV vaccine was 50.4%. The mean score of willingness to be vaccinated was 34.96 ± 7.187, and the details of willingness to be vaccinated for each entry are shown in Table 5.

**Table 5:** HPV vaccination willingness of college students in tertiary institutions.

Item	Number of negative attitudes (%)
Are you interested in learning about the HPV vaccine	511 (36.82)
Do you believe in the preventive effects of the HPV vaccine?	337 (24.28)
Do you believe in the safety of the HPV vaccine?	368 (26.51)
Your acceptance of the price of imported bivalent HPV vaccine (about 2,000)	672 (48.41)
Your acceptance of the price of imported quadrivalent HPV vaccine (about 3,000)	709 (51.08)
Your acceptance of the price of imported 9-valent HPV vaccine (about 4,000)	721 (51.95)
Would you like to be vaccinated against HPV	440 (31.70)
Would you like your friends and relatives to be vaccinated against HPV?	347 (25.00)
Would you be willing to get the HPV vaccine if it were included in the national health insurance?	300 (21.61)

According to the univariate analysis findings, there were variations found in university students’ willingness to receive the HPV vaccine depending on their sex, ethnicity, type of residence, major, monthly living expenses, parents’ literacy levels, family members’ employment status in the medical field, and family members’ cervical cancer status, as shown in Table 6. In particular, female students were found to have higher attitude scores than male students; students living in towns were found to have higher attitude scores than those living in rural areas; the attitude scores of medical-related majors were found to be higher than those of nonmedical-related majors; the willingness to be vaccinated was found to be lowest among students with monthly living expenses <1,000 RMB; the willingness to be vaccinated was found to be highest among students whose parents’ education level was specialized; the willingness to be vaccinated was found to be higher among students whose family members were in medically related occupations than among those whose parents were in nonmedically related occupations; and the willingness to be vaccinated was found to be highest among students whose family members were suffering from cervical cancer.

**Table 6:** Univariate analysis of HPV vaccination intention among college students with different demographic characteristics.

Participants		Mean score ± SD value	T/F value	*p* value	Cohen’s d/η^2^
Gender	Male	33.07 ± 6.990	−7.985	<0.001*	−0.440
	Female	36.16 ± 7.055	–	–	–
Ethnic group	Han	35.18 ± 7.129	5.062	0.006*	0.007
	Korean	33.49 ± 7.384	–	–	–
	Other nationality	35.47 ± 7.128	–	–	–
Town and country	Town	35.29 ± 7.326	2.883	0.004*	0.166
	Country	34.10 ± 6.749	–	–	–
Major	Medical major	35.68 ± 7.292	2.090	0.037*	0.131
	Non–medical major	34.73 ± 7.142	–	–	–
Grade	Freshman	35.20 ± 7.513	0.659	0.728	0.004
	Sophomore	34.53 ± 7.196	–	–	–
	Junior	35.30 ± 7.164	–	–	–
	Senior	34.91 ± 6.907	–	–	–
	Five–grade	33.36 ± 6.008	–	–	–
	First–year graduate students	35.40 ± 6.208	–	–	–
	Second–year graduate student	34.62 ± 5.852	–	–	–
	Third–year graduate student	34.48 ± 7.693	–	–	–
	Doctoral student	31.00 ± 15.232	–	–	–
Monthly living expenses	<1,000	30.74 ± 9.128	10.056	<0.001*	0.028
	1,000–1,999	34.39 ± 6.588	–	–	–
	2,000–2,999	35.82 ± 7.060	–	–	–
	3,000–3,999	37.01 ± 7.679	–	–	–
	≥4,000	34.46 ± 9.968	–	–	–
Father’s education level	Illiteracy	31.36 ± 11.011	4.658	<0.001*	0.020
	Secondary school	34.26 ± 6.707	–	–	–
	Junior high school	34.40 ± 6.650	–	–	–
	Senior high school	34.77 ± 7.074	–	–	–
	Specialized training school	36.88 ± 6.617	–	–	–
	Undergraduate	35.70 ± 7.570	–	–	–
	Graduate student and above	34.10 ± 9.244	–	–	–
Mother’s education level	Illiteracy	31.46 ± 9.736	4.754	<0.001*	0.020
	Secondary school	33.76 ± 6.644	–	–	–
	Junior high school	35.00 ± 6.662	–	–	–
	Senior high school	34.43 ± 7.081	–	–	–
	Specialized training school	36.23 ± 6.648	–	–	–
	Undergraduate	36.23 ± 7.644	–	–	–
	Graduate student and above	35.05 ± 9.441	–	–	–
Family members in medical–related professions	Yes	36.49 ± 7.185	4.086	<0.001*	0.271
	No	34.56 ± 7.137	–	–	–
Family member with cervical cancer	Yes	36.00 ± 8.542	5.256	0.005*	0.008
	No	35.13 ± 7.033	–	–	–
	Currently unknown	32.98 ± 8.094	–	–	–
Sexually active	Yes	34.71 ± 7.113	−0.588	0.556	−0.042
	No	35.01 ± 7.204	–	–	–

*p < 0.05.

Logistic regression analysis of the variables that were significantly different in the univariate analysis of HPV vaccination willingness revealed a significant positive relationship between females and the willingness to be actively vaccinated with the HPV vaccine (OR = 2.242, 95% CI = 1.777–2.829). Compared with the medically related population, the nonmedically related population was found to have an inverse relationship with the willingness to vaccinate (OR = 0.744, 95% CI = 0.570–0.970). In addition, compared with the population with monthly living expenses <1,000 RMB, the population with monthly living expenses of 2,000–2,999 RMB and 3,000–3,999 RMB were found to have a positive relationship with the willingness to receive the vaccination (OR = 1.926, 95% CI = 1.005–3.689; OR = 3.245, 95% CI = 1.488–7.077). Finally, having no family member in a medically related occupation was inversely associated with vaccination intentions (OR = 0.660, 95% CI = 0.493–0.883). All the above results were statistically significant, as shown in Table 7.

**Table 7:** Multifactorial logistic regression analysis of HPV vaccination willingness among college students in higher education.

Participants		*β* value	SE value	Wald χ^2^ value	*p* value	OR value (95%CI)
Gender	Male	–	–	–	–	1.00
	Female	0.807	0.119	46.354	<0.001*	2.242 (1.777 ~ 2.829)
Town and country	Town	–	–	–	–	1.00
	Country	−0.057	0.144	0.157	0.692	0.944 (0.712 ~ 1.253)
Major	Medical major	–	–	–	–	1.00
	Non–medical major	−0.296	0.135	4.777	0.029*	0.744 (0.570 ~ 0.970)
Monthly living expenses	<1,000	–	–	–	–	1.00
	1,000–1,999	0.616	0.325	3.584	0.058	1.851 (0.979 ~ 3.501)
	2,000–2,999	0.655	0.332	3.903	0.048*	1.926 (1.005 ~ 3.689)
	3,000–3,999	1.177	0.398	8.755	0.003*	3.245 (1.488 ~ 7.077)
	≥4,000	0.859	0.45	3.637	0.057	2.361 (0.976 ~ 5.708)
Father’s education level	Illiteracy	–	–	–	–	1.00
	Secondary school	−0.263	0.573	0.21	0.647	0.769 (0.250 ~ 2.364)
	Junior high school	−0.135	0.561	0.058	0.81	0.874 (0.291 ~ 2.625)
	Senior high school	−0.301	0.575	0.275	0.6	0.740 (0.240 ~ 2.283)
	Specialized training school	0.073	0.594	0.015	0.902	1.076 (0.336 ~ 3.443)
	Undergraduate	0.038	0.593	0.004	0.948	1.039 (0.325 ~ 3.322)
	Graduate student and above	−0.8	0.685	1.364	0.243	0.450 (0.118 ~ 1.720)
Mother’s education level	Illiteracy	–	–	–	–	1.00
	Secondary school	−0.086	0.491	0.031	0.861	0.917 (0.350 ~ 2.401)
	Junior high school	0.462	0.48	0.924	0.336	1.587 (0.619 ~ 4.070)
	Senior high school	0.411	0.497	0.683	0.408	1.508 (0.569 ~ 3.993)
	Specialized training school	0.601	0.519	1.345	0.246	1.825 (0.660 ~ 5.042)
	Undergraduate	0.662	0.52	1.62	0.203	1.938 (0.700 ~ 5.369)
	Graduate student and above	0.857	0.629	1.856	0.173	2.355 (0.687 ~ 8.078)
Family member with cervical cancer	Yes	–	–	–	–	1.00
	No	−0.150	0.396	0.143	0.705	0.861 (0.396 ~ 1.870)
	Currently unknown	−0.645	0.438	2.168	0.141	0.525 (0.222 ~ 1.238)
Family members in medical–related professions	Yes	–	–	–	–	1.00
	No	−0.416	0.149	7.828	0.005*	0.660 (0.493 ~ 0.883)

*p < 0.05.

#### 3.2.3 HPV vaccine-related behaviors of college students

The total positive behavior rate of college students toward the HPV vaccine was found to be 40.0%, with a mean score of 1.895 ± 1.456 for related behaviors, and only 25.43% (353) of those surveyed had already been vaccinated against HPV. A total of 62.18% (863) of the students said that they would publicize and recommend the HPV vaccine to others, and 52.81% (733) of the students said that they had inquired about the HPV vaccine; however, only 49.06% (681) had previously participated in HPV vaccine-related publicity activities (listening to lectures, watching videos).

The results of the univariate analysis revealed differences in the positive behavioral dimensions of the HPV vaccine among college students of different genders, types of residence, majors, grades, monthly living expenses, parental literacy, family members’ status in medically related occupations, family members’ status of cervical cancer, and sexual behavior, as shown in Table 8. Logistic regression analysis of the variables that exhibited significant differences in the univariate analysis indicated that females were positively correlated with favorable HPV vaccine behavior (OR = 2.687, 95% CI = 2.088–3.457), and nonmedically related professions were found to be inversely associated with positive vaccination behavior (OR = 0.579, 95% CI = 0.442–0.759). In addition, compared with the presence of family members in medically related occupations, those without family members in medically related occupations were inversely associated with positive vaccination behavior (OR = 0.693, 95% CI = 0.518–0.926). Compared with those who had family members with cervical cancer, those who had no family members with cervical cancer and those who were unsure about the presence of cervical cancer were found to be inversely associated with positive vaccination behavior (OR = 0.363, 95% CI = 0.160–0.824; OR = 0.346, 95% CI = 0.140–0.852). Finally, those who had not had sexual intercourse were found to have an inverse relationship positive vaccination behavior (OR = 0.651, 95% CI = 0.470–0.902). The results are shown in Table 9.

**Table 8:** Univariate analysis of HPV vaccine-related behaviors among college students with different demographic characteristics.

Participants		Number of positive behaviors (%)	χ^2^	*p*	Partial eta squared
Gender	Male	149 (27.64)	56.554	<0.001*	0.041
	Female	407 (47.94)	–	–	–
Ethnic group	Han	429 (30.91)	0.399	0.819	0.000
	Korean	77 (5.55)	–	–	–
	Other nationality	50 (3.60)	–	–	–
Town and country	Town	422 (42.20)	6.838	0.009*	0.005
	Country	134 (34.54)	–	–	–
Major	Medical major	172 (51.81)	25.089	<0.001*	0.018
	Non–medical major	384 (36.36)	–	–	–
Grade	Freshman	172 (41.15)	17.299	0.027*	0.012
	Sophomore	123 (34.07)	–	–	–
	Junior	117 (40.77)	–	–	–
	Senior	40 (36.04)	–	–	–
	Five–grade	8 (57.14)	–	–	–
	First–year graduate students	50 (50.00)	–	–	–
	Second–year graduate student	22 (42.31)	–	–	–
	Third–year graduate student	20 (50.00)	–	–	–
	Doctoral student	4 (80.00)	–	–	–
Monthly living expenses	<1,000	16 (27.59)	17.534	0.002*	0.013
	1,000–1,999	238 (36.17)	–	–	–
	2,000–2,999	234 (43.41)	–	–	–
	3,000–3,999	46 (52.87)	–	–	–
	≥4,000	22 (47.83)	–	–	–
Father’s education level	Illiteracy	9 (36.00)	28.982	<0.001*	0.021
	Secondary school	42 (25.77)	–	–	–
	Junior high school	153 (36.34)	–	–	–
	Senior high school	116 (40.70)	–	–	–
	Specialized training school	93 (48.95)	–	–	–
	Undergraduate	114 (46.72)	–	–	–
	Graduate student and above	29 (48.33)	–	–	–
Mother’s education level	Illiteracy	12 (34.29)	23.416	<0.001*	0.017
	Secondary school	65 (31.10)	–	–	–
	Junior high school	148 (36.36)	–	–	–
	Senior high school	115 (40.49)	–	–	–
	Specialized training school	78 (44.83)	–	–	–
	Undergraduate	105 (47.51)	–	–	–
	Graduate student and above	33 (56.90)	–	–	–
Family members in medical–related professions	Yes	149 (51.92)	21.191	<0.001*	0.015
	No	407 (36.97)	–	–	–
Family member with cervical cancer	Yes	20 (66.67)	10.583	0.005*	0.008
	No	495 (39.98)	–	–	–
	Currently unknown	41 (34.17)	–	–	–
Sexually active	Yes	110 (47.62)	6.599	0.010*	0.005
	No	446 (38.55)	–	–	–

*p < 0.05.

**Table 9:** Multifactorial logistic regression analysis of HPV vaccine-related behaviors among college students in higher education.

Participants		*β* value	SE value	Wald χ^2^ value	*p* value	OR value (95%CI)
Gender	Male	–	–	–	–	1.00
	Female	0.988	0.129	58.981	<0.001*	2.687 (2.088 ~ 3.457)
Town and country	Town	–	–	–	–	1.00
	Country	0.079	0.15	0.274	0.601	1.082 (0.806 ~ 1.453)
Major	Medical major	–	–	–	–	1.00
	Non–medical major	−0.546	0.138	15.597	<0.001*	0.579 (0.442 ~ 0.759)
Grade	Freshman	–	–	–	–	1.00
	Sophomore	−0.254	0.158	2.574	0.109	0.776 (0.569 ~ 1.058)
	Junior	0.072	0.166	0.187	0.666	1.075 (0.776 ~ 1.489)
	Senior	−0.146	0.236	0.384	0.536	0.864 (0.544 ~ 1.373)
	Five–grade	0.344	0.584	0.346	0.556	1.41 (0.449 ~ 4.430)
	First–year graduate students	0.395	0.243	2.636	0.104	1.485 (0.921 ~ 2.393)
	Second–year graduate student	−0.049	0.317	0.023	0.878	0.953 (0.511 ~ 1.775)
	Third–year graduate student	0.031	0.356	0.008	0.931	1.031 (0.514 ~ 2.070)
	Doctoral student	1.158	1.189	0.949	0.330	3.183 (0.310 ~ 32.715)
Monthly living expenses	<1,000	–	–	–	–	1.00
	1,000–1,999	0.314	0.338	0.864	0.352	1.369 (0.706 ~ 2.655)
	2,000–2,999	0.408	0.345	1.399	0.237	1.503 (0.765 ~ 2.954)
	3,000–3,999	0.699	0.405	2.982	0.084	2.011 (0.910 ~ 4.444)
	≥4,000	0.286	0.465	0.379	0.538	1.331 (0.535 ~ 3.310)
Father’s education level	Illiteracy	–	–	–	–	1.00
	Secondary school	−0.546	0.603	0.821	0.365	0.579 (0.178 ~ 1.888)
	Junior high school	−0.019	0.587	0.001	0.974	0.981 (0.310 ~ 3.100)
	Senior high school	0.166	0.601	0.076	0.782	1.181 (0.363 ~ 3.836)
	Specialized training school	0.451	0.62	0.53	0.467	1.570 (0.466 ~ 5.292)
	Undergraduate	0.254	0.619	0.168	0.682	1.289 (0.383 ~ 4.332)
	Graduate student and above	−0.442	0.714	0.382	0.536	0.643 (0.159 ~ 2.607)
Mother’s education level	Illiteracy	–	–	–	–	1.00
	Secondary school	0.143	0.514	0.078	0.78	1.154 (0.422 ~ 3.158)
	Junior high school	0.031	0.504	0.004	0.951	1.032 (0.384 ~ 2.772)
	Senior high school	0.130	0.520	0.063	0.802	1.139 (0.411 ~ 3.159)
	Specialized training school	0.039	0.543	0.005	0.943	1.040 (0.359 ~ 3.012)
	Undergraduate	0.187	0.542	0.119	0.730	1.205 (0.417 ~ 3.484)
	Graduate student and above	1.092	0.653	2.793	0.095	2.98 (0.828 ~ 10.721)
Family member with cervical cancer	Yes	–	–	–	–	1.00
	No	−0.367	0.148	6.133	0.013*	0.693 (0.518 ~ 0.926)
	Currently unknown	–	–	–	–	1.00
Sexually active	Yes	−1.012	0.418	5.865	0.015*	0.363 (0.160 ~ 0.824)
	No	−1.063	0.46	5.329	0.021*	0.346 (0.140 ~ 0.852)
Family member with cervical cancer	Yes	–	–	–	–	1.00
	No	−0.429	0.166	6.653	0.010*	0.651 (0.470 ~ 0.902)

*p < 0.05.

#### 3.2.4 Status of the health belief model

The evaluation of participants in this study utilized the five dimensions of the health belief model: perceived benefit, perceived severity, self-efficacy, perceived handicap, and perceived susceptibility.

The scores for each dimension of willingness to receive the vaccination were in the same order as the overall perceptions. The scores of perceived severity (3.58 ± 1.09), self-efficacy (3.32 ± 1.00), and perceived benefit (3.86 ± 1.14) were greater for those with positive vaccination attitudes than for those with negative attitudes (3.38 ± 0.76; 3.13 ± 0.65; and 3.51 ± 0.78, respectively), and the differences were statistically significant (all *p* < 0.05). The score of negative vaccination willingness (3.14 ± 0.73) was greater than that of cheerful vaccination willingness (2.96 ± 1.08) for the perceptual obstructive dimension, and the difference was statistically significant (*p* < 0.05). There was no statistically significant difference found between the two scores on the perceived susceptibility dimension (*p* > 0.05) (see Table 10).

**Table 10:** Comparison of health belief dimension scores of different HPV vaccination intendants.

	Overall score	Negative intentions score (<36)	Active volunteer score (≥36)	T/F	*p*
Susceptibility	2.79 ± 1.04	2.76 ± 0.89	2.83 ± 1.16	1.217	0.224
Perceived severity	3.48 ± 0.95	3.38 ± 0.76	3.58 ± 1.09	3.915	<0.001*
Perceptual disability	3.05 ± 0.93	3.14 ± 0.73	2.96 ± 1.08	−3.615	<0.001*
Self-efficacy	3.23 ± 0.85	3.13 ± 0.65	3.32 ± 1.00	4.173	<0.001*
Perceived benefits	3.69 ± 1.00	3.51 ± 0.78	3.86 ± 1.14	6.680	<0.001*

*p < 0.05.

### 3.3 Confirmatory factor analysis

This research investigated six variables: willingness to vaccinate, perceived susceptibility, perceived severity, perceived disability, self-efficacy, and perceived benefit. The model had a KMO value of 0.915 and a Bartlett test of sphericity value of <0.01, which was statistically significant at the *α* = 0.05 level, indicating that it was possible to perform a factor analysis. Perceived severity (with five observations) was the exogenous latent variable in this survey. Perceived susceptibility (with five observations), perceived handicap (with two observations), self-efficacy (with three observations), perceived benefit (with three observations), and willingness to be vaccinated (with six observations) were the endogenous latent variables. A validated factor analysis was conducted via AMOS 24.0 software. The mean of the six factors corresponding to the variance extraction (AVE) values was more significant than 0.5. The combined reliability (CR) values were higher than 0.7, and the current data had good convergent validity.

### 3.4 Construction of structural equation modeling

**Figure 1:**
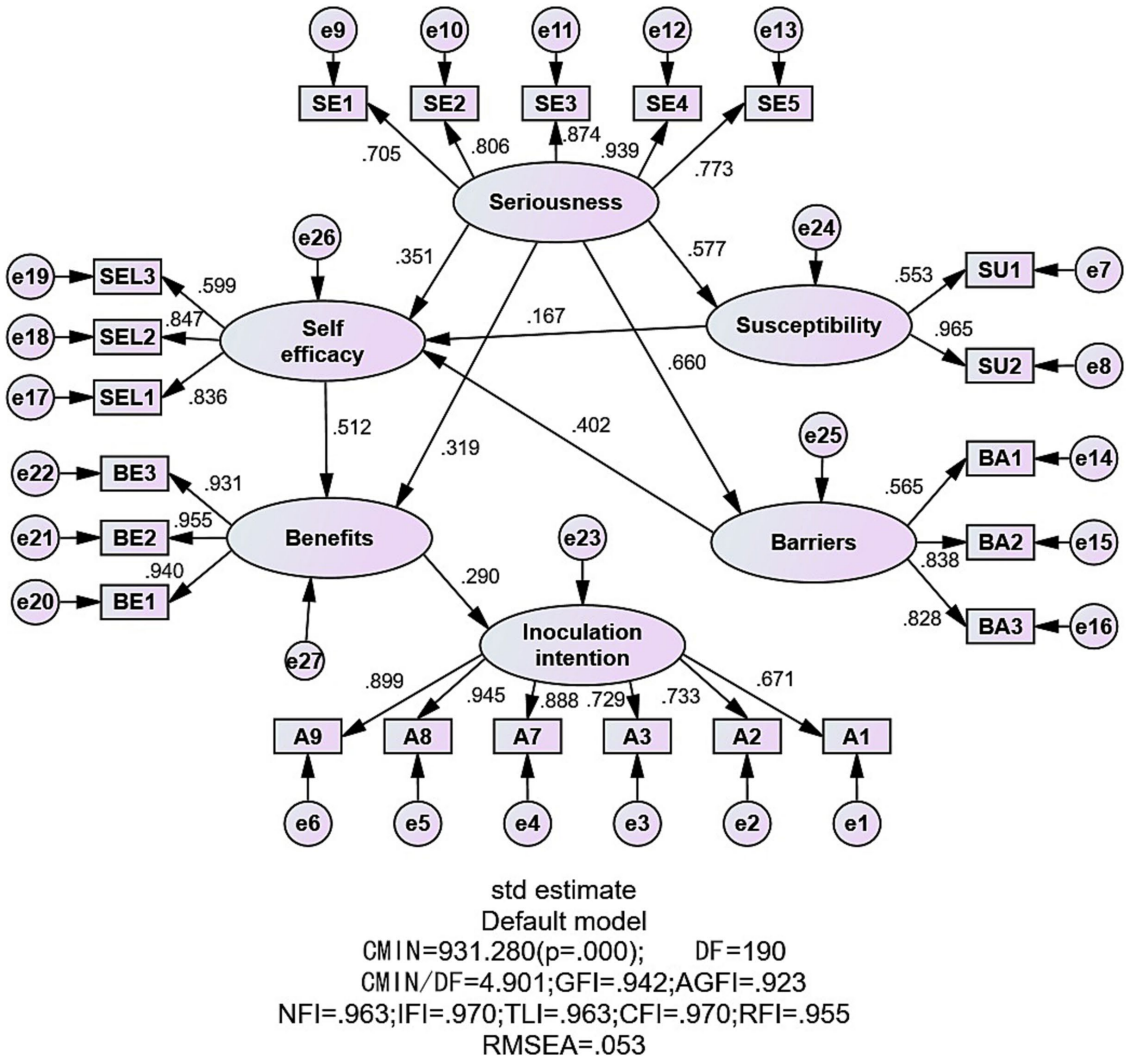
Structural equation modeling of factors influencing college students’ attitudes toward HPV vaccination.

Based on the theoretical framework of the health belief model, the six factors obtained from the exploratory factor analysis were used as latent variables to construct a structural equation model. The model was continuously revised and fitted via the excellent likelihood method until the constructed model fit the measurement data better. The model was finalized as shown in Figure 1. The modified model fit indices all met the corresponding reference standards, χ^2^/df = 4.901 (<5); GFI = 0.942, AGFI = 0.923, NFI = 0.963, IFI = 0.970, TLI = 0.963, CFI = 0.970, RFI = 0.955 (All>0.90); RMSEA = 0.053 (<0.08). The model was subjected to path analysis; the results are shown in Table 11. The factor that directly influences the willingness to vaccinate against HPV is the perceived benefit, which increases by 0.29 standard units for every 1 standard unit increase in perceived benefit.

**Table 11:** Structural equation modeling path coefficients.

Pathway			Unstandardized path coefficient estimates	SE	CR	*p* value	Standardized path coefficient estimates
Susceptibility	<---	Seriousness	0.502	0.038	13.539	<0.001*	0.577
Barriers	<---	Seriousness	0.562	0.034	16.357	<0.001*	0.660
Benefits	<---	Seriousness	0.228	0.038	6.020	<0.001*	0.167
Self_efficacy	<---	Seriousness	0.559	0.052	10.749	<0.001*	0.402
Self_efficacy	<---	Susceptibility	0.416	0.044	9.420	<0.001*	0.351
Self_efficacy	<---	Barriers	0.566	0.041	13.783	<0.001*	0.512
Benefits	<---	Self_efficacy	0.419	0.046	9.193	<0.001*	0.319
Inoculation_intention	<---	Benefits	0.203	0.020	10.169	<0.001*	0.290

*p < 0.05.

In addition, perceived severity does not directly affect willingness to vaccinate but can indirectly affect perceived susceptibility, perceived handicap, self-efficacy, and perceived benefit. Based on the identified model, the mediating roles of perceived ease of use, perceived barriers, self-efficacy, and perceived benefits in the model were analyzed via the bias-corrected bootstrap procedure, with 5,000 repetitive samples and mediation effect tests and confidence interval estimation performed, which indicated a significant indirect effect as the 95% confidence interval did not include zero. As shown in Table 12, the indirect and total effects of perceived severity on willingness to vaccinate had *p* values <0.05, with perceived benefit (95% CI = 0.059–0.131) playing a partial mediating role and self-efficacy-perceived benefit (95% CI = 0.033–0.082) and perceived susceptibility-self-efficacy-perceived benefit (95% CI = 0.008–0.023) and perceived handicap-self-efficacy-perceived benefit (95% CI = 0.028–0.056) acting as chain mediators.

**Table 12:** Intermediary test results.

Typology	Intermediary path	Standardized estimates	Efficiency ratio	SE	Boot 95%CI	*p*
Intermediary effect	Seriousness – Self_efficacy – Benefits – Inoculation_intention	0.052	26.26%	0.012	0.033–0.082	<0.001*
	Seriousness – Benefits – Inoculation_intention	0.092	46.46%	0.018	0.059–0.131	<0.001*
	Seriousness – Susceptibility – Self_efficacy – Benefits – Inoculation_intention	0.014	7.07%	0.004	0.008–0.023	<0.001*
	Seriousness – Barriers – Self_efficacy – Benefits – Inoculation_intention	0.039	19.70%	0.007	0.028–0.056	<0.001*
Total effect	–	0.198	100.00%	–	–	–

*p < 0.05.

## 4 Discussion

### 4.1 College students’ knowledge, willingness to vaccinate, and behavior regarding HPV and the HPV vaccine

In recent years, cervical cancer has been a significant public health problem that threatens women’s health globally, and the usage of the HPV vaccine is the key to preventing cervical cancer. Thus, our study conducted an in-depth investigation of the current status of HPV vaccination among college students in ethnic minority areas.

The results of this study revealed that the participants’ overall knowledge about HPV was good. Most of the students had a good grasp of vaccine-related knowledge but poor knowledge about the symptoms of HPV infection and the high-risk types of the virus. The reason for this phenomenon may be that the type of information disseminated on the internet is mainly at the level of vaccination and modes of infection, with a relative scarcity of pertinent academic knowledge being disseminated; thus, the general public does not have sufficient awareness of more in-depth academic knowledge (36, 37). Studies have shown that overall knowledge is greater in women than in men, which is consistent with the findings of many national studies (38, 39); this difference may stem from a greater level of concern about health issues among women. Considering that HPV infection is associated mainly with cervical cancer, which occurs predominantly in women, this may explain the greater level of concern for such knowledge among women. The results revealed that students of Korean ethnicity had a lower level of positive HPV knowledge than did students of Han Chinese ethnicity. This result may be related to differences in the ethnic cultures. Some studies have suggested that cultural factors may hinder the discussion and acceptance of HPV (40). Moreover, China’s publicity for HPV-related knowledge is generally in the form of the Chinese language, which is a nonnative language for Korean college students; thus, Korean college students may have poorer comprehension level of the language, which may also be a potential reason for the significantly lower knowledge rate of Koreans than that of Han Chinese regarding HPV knowledge. The knowledge rate of college students in medical-related majors was found to be higher than that of students in nonmedical-related majors, possibly because medical majors have more corresponding access to information, which can make it easier for them to learn the corresponding medical-related knowledge; this is consistent with the results of other studies (41, 42). Compared with monthly living expenses of <1,000 RMB, living expenses of 2,000–2,999 RMB had a positive effect on HPV knowledge among college students; previous evidence (43) has suggested that family income is an essential factor influencing young people’s acceptance of the HPV vaccine, which suggests that better-off students may be more concerned about their health due to family support and richer educational resources and are more likely to have access to HPV-related information sources and thus have higher HPV knowledge rates than their counterparts. Therefore, it is essential to focus on specific groups, such as men, Korean individuals, people who do not work in medicine, and people whose monthly living costs are less than 1,000 RMB, when making health education and promotion plans to help these individuals learn more about HPV.

The survey results revealed that the rate of cheerful willingness of college students to be vaccinated against HPV was moderate. Approximately half of these college students had negative attitudes about the import prices of HPV vaccines (including bivalent, quadrivalent, and nine-valent). A previous study of Chinese female college students revealed that the vaccine price is an essential factor affecting vaccination rates (44), which suggests that high vaccine prices may be one of the reasons hindering college students’ willingness to be actively vaccinated. In addition, college students may have less knowledge about the importance and long-term benefits of the HPV vaccine, and a lack of proper understanding of the price of the vaccine investment may also contribute to the low rate of positive intentions. It was found that females had significantly higher positive willingness scores than males and were 2.242 times more likely to be positively willing to be vaccinated with the HPV vaccine than males were; these results show that female students are more interested in vaccination, which is in line with the results of other studies (45). The fact that nonmedical students lack a background in medical knowledge and do not have easy access to pertinent vaccine information (41), which in turn causes them to have doubts about the efficacy and safety of vaccines, may help explain why having a nonmedical major negatively affects one’s positive intention. The study indicated that college students with higher living expenses (2,000–2,999 RMB and 3,000–3,999 RMB per month) had a greater willingness to be vaccinated against HPV than did those with living expenses of <1,000 RMB; this result that is consistent with findings from other studies (31). There is existing evidence that the cost of vaccination is one of the most common barriers to receiving the vaccine (46). In the present study, the probability of positive vaccination intention was found to be significantly lower among those with no family members in medically related occupations than among those with family members in medically related occupations. Healthcare workers and family members are the most influential sources of information (47); thus, family members who work in medically related occupations may be trusted more highly because of their medical background and professional status. The information and opinions that these individuals convey may have a more positive effect on family members. Therefore, improving attitudes toward immunization interventions in different social roles is necessary.

The results revealed that the examined college students had a poor rate of total positive behavior toward the HPV vaccine, with only 25.43% having already received the HPV vaccine. Among them, women’s positive behavior regarding the HPV vaccine was 2.687 times greater than men’s positive behavior. Previous studies have noted that men are less receptive to the 9-valent HPV vaccine, with approximately 54.95% of them expressing reluctance to receive it (48). Sociocultural factors related to gender may be responsible for this phenomenon. Given that cervical cancer is the primary health risk women may encounter, men typically perceive a lower risk of HPV infection than women, leading to a generally lower willingness and behavior for vaccination. Further research is necessary to validate this relationship, even though these results align with sociocultural factors associated with gender differences. The probability of positive behavior was found to be significantly lower among those who did not have or were unsure if they had friends of family members with cervical cancer than among college students with friends or relatives who had cervical cancer. It has been shown previously that having a relative or friend with cervical cancer is a positive influencing factor for HPV vaccination (31), which suggests that the probability of adopting positive behaviors is more significant when its severity is recognized. The results indicated that sexually active college students are more inclined to engage in positive behaviors, such as vaccination, aligning with the conclusions of other studies (27, 31). Other studies have indicated that women who have never been sexually active are less likely to receive vaccinations than sexually active women are (49), which may suggest that receiving sex education may be an essential way to increase positive behaviors. Therefore, there is a need to emphasize the importance of vaccination through such education.

### 4.2 Influence of health belief patterns on positive vaccination intentions

We used the situation of university students’ willingness to be vaccinated in ethnic minority areas in China, combined it with the health belief model, and developed structural equation modeling to explore the pathways influencing the willingness to receive HPV vaccination. The current study revealed that the health belief of college students is in a good state and that there is a positive association between the level of health belief and the willingness to receive HPV vaccination; this means that those who have a cheerful desire to receive vaccination also have stronger health beliefs.

According to this study, perceived benefits directly impact willingness to vaccinate, and both factors change in the same direction. As a result, college students are more likely to demonstrate a cheerful desire to vaccinate, and the perceived benefits are more substantial (50). One previous study found that a video-based behavioral intervention effectively increased parents’ and children’s willingness to be vaccinated against HPV (51). Therefore, to increase the willingness to vaccinate, we suggest increasing the efforts of public education and increasing the forms of video education, including health talks, the production of multilingual promotional videos, and media reports, to convey upbeat messages to the public, all of which will augment the public’s perception of the advantages of the HPV vaccine, thereby elevating the propensity to vaccinate.

Perceived severity indirectly affects the willingness to vaccinate through other factors; thus, increasing the perceived severity of college students can effectively increase the willingness to vaccinate. Notably, increasing an individual’s perception of disease severity can increase their motivation to protect, thereby promoting willingness to change behavior (30). In addition, it has been shown that HPV can cause almost all types of cervical cancer, as well as many cancers at other anatomical sites in both men and women (52); thus, HPV education should focus on highlighting the high prevalence and severity of HPV-associated diseases to increase the level of perceived risk for educated individuals.

This study revealed that perceived susceptibility mediates one’s willingness to be vaccinated and influences one’s willingness to vaccinate in the same direction. That is, the stronger the perceived susceptibility is, the greater the willingness to vaccinate is; this finding is similar to the results found by other studies (53). The greater the individual’s perceived risk of HPV infection is, the greater their confidence in the efficacy of the HPV vaccine is, and the greater their willingness to be vaccinated is (54). These findings suggest that increasing college students’ perceived risk of HPV infection facilitates their willingness to vaccinate. Consequently, healthcare professionals should be motivated to proactively educate the public regarding HPV and its vaccine, as well as to advocate for familial education, encouraging parents to engage in more dialogues with their children about the risks associated with HPV and the significance of vaccination to enhance the willingness to receive the vaccine.

The study’s results showed that perceived barriers mediate the effect on willingness to vaccinate, such that the more potent the perception of barriers is, the lower the level of willingness to vaccinate is; this is consistent with the results found by other studies (55). Notably, cost, safety, and side effects have been frequently reported as significant barriers to accessing vaccines (46). Other studies have emphasized that despite the public awareness of the safety and efficacy of HPV vaccines, these factors remain essential barriers to vaccine acceptance (56). This highlights the need to convey HPV vaccine safety data and relevant research results to the public through various communication channels, such as popularization activities, special lectures, and social media, which will help the public dispel misconceptions about the vaccine. In addition, the government should consider introducing a subsidy policy for HPV vaccines to lower the cost of vaccination so that people of the right age from low-income families can also afford the cost of vaccination, thus lowering the barriers to vaccination.

In the health belief model, self-efficacy positively mediated HPV vaccination intentions, similar to previous findings (57). It suggests that increasing college students’ self-efficacy will help increase their willingness to vaccinate by providing a deeper understanding of the convenience and effectiveness of vaccination. Improving self-efficacy can be done through knowledge about HPV, peer education, or by attending relevant seminars, getting advice and support from healthcare practitioners, and increasing confidence in vaccination.

Despite this study’s theoretical value and practical significance, several limitations remain. First, the sample chosen for this study included only a subset of students enrolled at a comprehensive public university, rather than the entire population of college students at the institution. Therefore, the ability of the findings to accurately reflect the perceptions and behaviors of a larger population of college students may be controversial. In the future, the sample size can be enlarged on the basis of the current study so that the study can be conducted from a broader perspective. Second, the online questionnaire format used in this study may have led to some recall bias among the participants, which may have affected the accuracy of the findings. Finally, this study has a cross-sectional research design that uses structural equation modeling to demonstrate direct and indirect effects between variables. However, it focuses primarily on modeling relationships, which does not allow causal inferences to be made.

## 5 Conclusion

College students in China’s ethnic minority areas were found to have better knowledge about HPV and its vaccine but showed an average level of willingness to be vaccinated and a lower proportion of positive behaviors. From the perspective of the health belief model, perceived benefits were found to have a significant positive direct effect on the willingness to vaccinate, and perceived severity were found to have a positive indirect effect on the desire to vaccinate. Furthermore, the chain mediating effects of perceived susceptibility, perceived impediment, and self-efficacy were found to play an essential role in increasing the willingness to vaccinate. On this basis, it is recommended that schools and departments implement targeted interventions for special populations; strengthen publicity and education efforts; and improve college students’ level of knowledge, willingness to be vaccinated, and behavior toward HPV vaccination to promote HPV vaccination and thus help reduce the potential health hazards of HPV.

## Data Availability

The original contributions presented in the study are included in the article/supplementary material, further inquiries can be directed to the corresponding author.
